# Supplementary material for: Network-Based Integration of Multi-Omics Data Identifies the Determinants of miR-491-5p Effects
Source: Cancers (Basel). 2021 Aug 5;13(16):3970. doi: 10.3390/cancers13163970 (PMC8393872; doi:10.3390/cancers13163970)

(A)

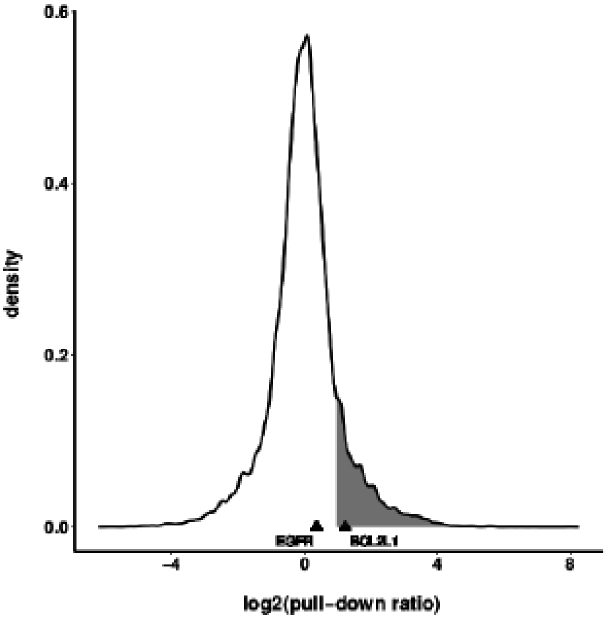


(B)

| Considered experiments | Number of genes |
|---|---|
| Pull-Down + Transcriptome + Proteome | 3,647 genes |
| Pull-Down + Transcriptome | 3,363 genes |
| Pull-Down + Proteome | 3,097 genes |
| Transcriptome + Proteome | 1,037 genes |
| Pull-Down | 2,754 genes |
| Transcriptome | 739 genes |
| Proteome | 371 genes |

(C)

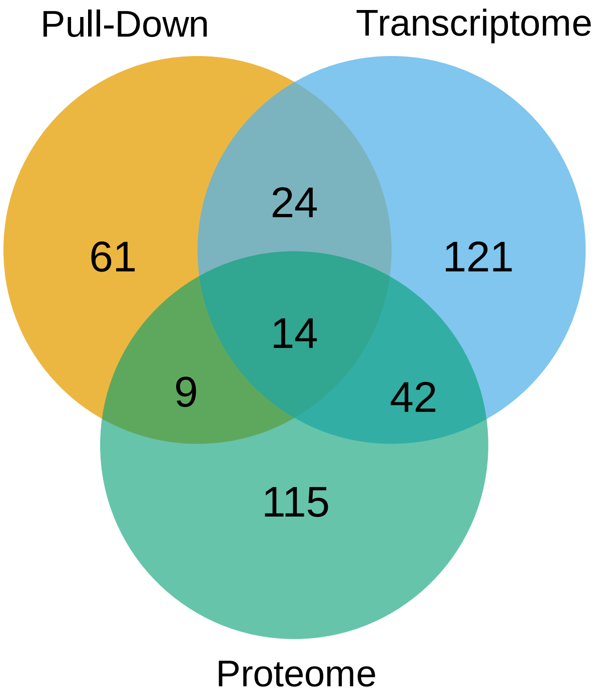

Supplement: Supplementary file 1 [file cancers-13-03970-s001.zip › Figure S1.pdf]
