# Supplementary material for: Network-Based Integration of Multi-Omics Data Identifies the Determinants of miR-491-5p Effects
Source: Cancers (Basel). 2021 Aug 5;13(16):3970. doi: 10.3390/cancers13163970 (PMC8393872; doi:10.3390/cancers13163970)

(A)

Pull-Down + Transcriptome + Proteome	Pull-Down	Transcriptome	Proteome
REGULATION OF TRANSPORT	REGULATION OF TRANSPORT	REGULATION OF INTRACELLULAR SIGNAL TRANSDUCTION	CATABOLIC PROCESS
REGULATION OF INTRACELLULAR SIGNAL TRANSDUCTION	HOMEOSTATIC PROCESS	POSITIVE REGULATION OF GENE EXPRESSION	PROTEIN LOCALIZATION
POSITIVE REGULATION OF RESPONSE TO STIMULUS	CHEMICAL HOMEOSTASIS	TISSUE DEVELOPMENT	ESTABLISHMENT OF PROTEIN LOCALIZATION
TISSUE DEVELOPMENT	POSITIVE REGULATION OF RESPONSE TO STIMULUS	POSITIVE REGULATION OF BIOSYNTHETIC PROCESS	CELLULAR CATABOLIC PROCESS
INTRACELLULAR SIGNAL TRANSDUCTION	BIOLOGICAL ADHESION	INTRACELLULAR SIGNAL TRANSDUCTION	SMALL MOLECULE METABOLIC PROCESS
POSITIVE REGULATION OF MOLECULAR FUNCTION	POSITIVE REGULATION OF MOLECULAR FUNCTION	EMBRYO DEVELOPMENT	ORGANONITROGEN COMPOUND METABOLIC PROCESS
PHOSPHATE CONTAINING COMPOUND METABOLIC PROCESS	REGULATION OF INTRACELLULAR SIGNAL TRANSDUCTION	POSITIVE REGULATION OF RESPONSE TO STIMULUS	SINGLE ORGANISM CATABOLIC PROCESS
POSITIVE REGULATION OF CELL COMMUNICATION	VESICLE MEDIATED TRANSPORT	PHOSPHATE CONTAINING COMPOUND METABOLIC PROCESS	MACROMOLECULAR COMPLEX ASSEMBLY
REGULATION OF PHOSPHORUS METABOLIC PROCESS	ION TRANSPORT	ORGAN MORPHOGENESIS	ESTABLISHMENT OF LOCALIZATION IN CELL
HOMEOSTATIC PROCESS	INTRACELLULAR SIGNAL TRANSDUCTION	POSITIVE REGULATION OF MOLECULAR FUNCTION	PROTEIN COMPLEX SUBUNIT ORGANIZATION
REGULATION OF MULTICELLULAR ORGANISMAL DEVELOPMENT	TISSUE DEVELOPMENT	REGULATION OF PHOSPHORUS METABOLIC PROCESS	REGULATION OF CELLULAR RESPONSE TO STRESS
RESPONSE TO OXYGEN CONTAINING COMPOUND	PROTEIN LOCALIZATION	POSITIVE REGULATION OF CELL COMMUNICATION	SINGLE ORGANISM BIOSYNTHETIC PROCESS
CELLULAR RESPONSE TO ORGANIC SUBSTANCE	ORGAN MORPHOGENESIS	REGULATION OF RESPONSE TO STRESS	PHOSPHATE CONTAINING COMPOUND METABOLIC PROCESS
REGULATION OF RESPONSE TO STRESS	NEGATIVE REGULATION OF MOLECULAR FUNCTION	REGULATION OF PROTEIN MODIFICATION PROCESS	REGULATION OF RESPONSE TO STRESS
PROTEIN LOCALIZATION	REGULATION OF MULTICELLULAR ORGANISMAL DEVELOPMENT	CELL DEVELOPMENT	RESPONSE TO OXYGEN CONTAINING COMPOUND
REGULATION OF PROTEIN MODIFICATION PROCESS	LIPID METABOLIC PROCESS	REGULATION OF TRANSCRIPTION FROM RNA POLYMERASE II PROMOTER	ORGANIC ACID METABOLIC PROCESS
EMBRYO DEVELOPMENT	POSITIVE REGULATION OF CATALYTIC ACTIVITY	REGULATION OF CELL DEATH	PROTEIN COMPLEX BIOGENESIS
POSITIVE REGULATION OF GENE EXPRESSION	NEGATIVE REGULATION OF RESPONSE TO STIMULUS	CELLULAR RESPONSE TO ORGANIC SUBSTANCE	POSITIVE REGULATION OF CELLULAR COMPONENT ORGANIZATION
IMMUNE SYSTEM PROCESS	REGULATION OF CELL PROLIFERATION	ANATOMICAL STRUCTURE FORMATION INVOLVED IN MORPHOGENESIS	LIPID METABOLIC PROCESS
ANATOMICAL STRUCTURE FORMATION INVOLVED IN MORPHOGENESIS	CELLULAR RESPONSE TO ORGANIC SUBSTANCE	CELL DEATH	VESICLE MEDIATED TRANSPORT

(B)

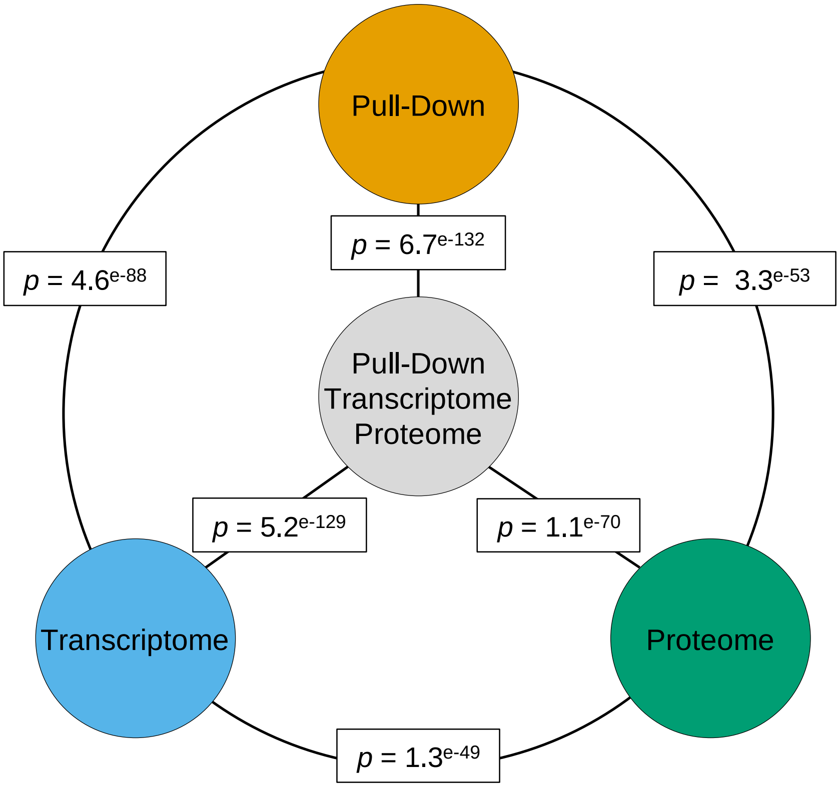

Supplement: Supplementary file 1 [file cancers-13-03970-s001.zip › Figure S2.pdf]
