# Supplementary material for: Network-Based Integration of Multi-Omics Data Identifies the Determinants of miR-491-5p Effects
Source: Cancers (Basel). 2021 Aug 5;13(16):3970. doi: 10.3390/cancers13163970 (PMC8393872; doi:10.3390/cancers13163970)

Raw Blots from Figure 5D

Original blots images are provided by the imager within a green frame.
Samples related specifically to this figure part are with legends, samples unrelated to this specific figure part which were imaged at the same on different membranes are legended as « other ».

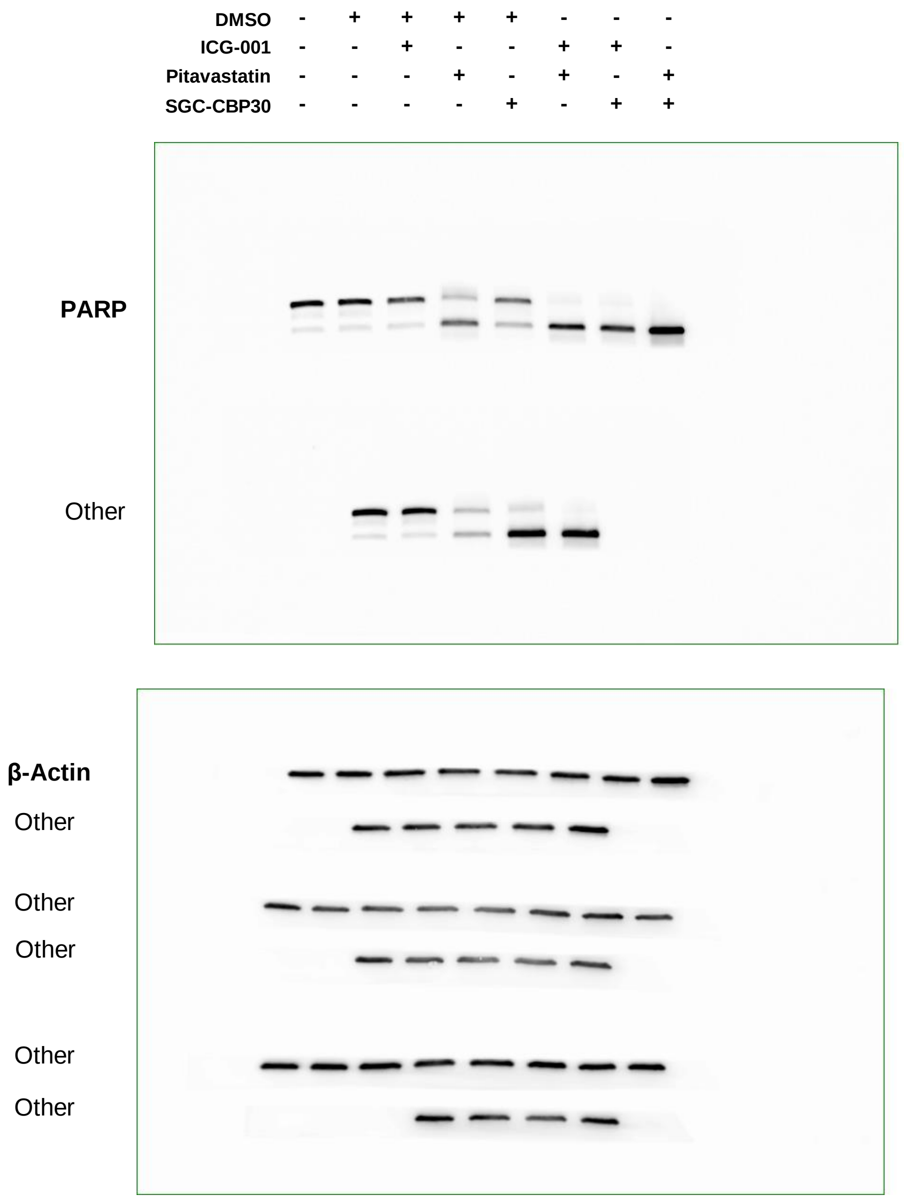

Raw Blots from Figure 5D

Original blots images are provided by the imager within a green frame.
Samples related specifically to this figure part are with legends, samples unrelated to this specific figure part which were imaged at the same on different membranes are legended as « other ».

**DMSO**	-	+	+	+	+	-	-	-
**ICG-001**	-	-	+	-	-	+	+	-
**Pitavastatin**	-	-	-	+	-	+	-	+
**SGC-CBP30**	-	-	-	-	+	-	+	+

Total C-3

Cleaved C-3

Other

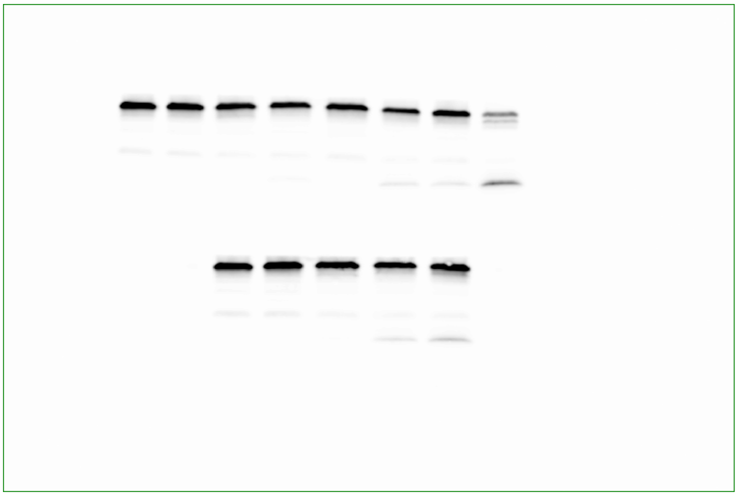

Total C-3 (longer exposure)

Cleaved C-3 (longer exposure)

Other

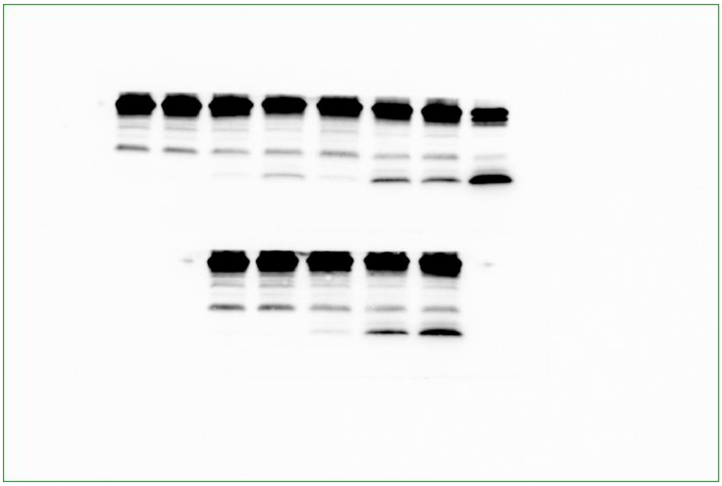

Supplement: Supplementary file 1 [file cancers-13-03970-s001.zip › Figure S4.pdf]
