# Supplementary material for: Network-Based Integration of Multi-Omics Data Identifies the Determinants of miR-491-5p Effects
Source: Cancers (Basel). 2021 Aug 5;13(16):3970. doi: 10.3390/cancers13163970 (PMC8393872; doi:10.3390/cancers13163970)
Supplement: Supplementary file 1 [file cancers-13-03970-s001.zip › supplementary_MMF_MV.pdf]

cancers

Supplementary

Network-based integration of multi-omics data identifies the determinants of miR-491-5p effects

Supplementary Materials

Figure S1. Number of selected genes considered for analyses.
(A) Distribution of transcripts pull-down enrichment ratio (log scale). Gray-colored section represents genes above 0.95 enrichment ratio (Log2) which was used as a cut-off. EGFR and BCL2L1 (Bcl-xL), previously demonstrated to be direct targets of miR-491-5p in IGROV1-R10 cells, are highlighted. (**B**) Number of genes from each experiment or combination of experiments which was considered for further analyses. (**C**) Overlapping genes from the three experiments.

(A)

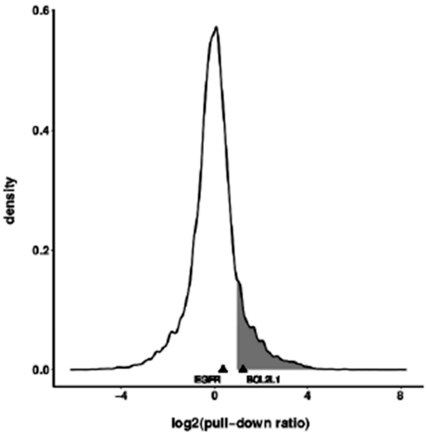

(B)

Considered experiments	Number of genes
Pull-Down + Transcriptome + Proteome	3,647 genes
Pull-Down + Transcriptome	3,363 genes
Pull-Down + Proteome	3,097 genes
Transcriptome + Proteome	1,037 genes
Pull-Down	2,754 genes
Transcriptome	739 genes
Proteome	371 genes

(C)

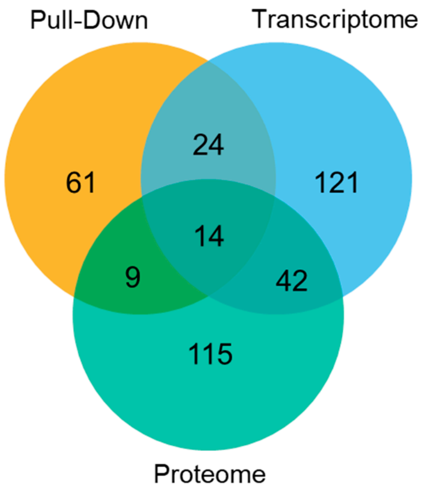

Figure S2. Combination of 3 experiments for GO-pathway analysis provides integrated results.
(A) Differentially expressed/enriched gene lists from single or combined experiments were analyzed according to GO-Biological Processes database. The top 20 most enriched terms are presented. (**B**) Overlaps of the 100 most enriched terms between individual experiments, and between each experiment and the combined analysis. Significance of the overlap was calculated with a hypergeometric test.

(A)

Pull-Down + Transcriptome + Proteome	Pull-Down	Transcriptome	Proteome
REGULATION OF TRANSPORT	REGULATION OF TRANSPORT	REGULATION OF INTRACELLULAR SIGNAL TRANSDUCTION	CATABOLIC PROCESS
REGULATION OF INTRACELLULAR SIGNAL TRANSDUCTION	HOMEOSTATIC PROCESS	POSITIVE REGULATION OF GENE EXPRESSION	PROTEIN LOCALIZATION
POSITIVE REGULATION OF RESPONSE TO STIMULUS	CHEMICAL HOMEOSTASIS	TISSUE DEVELOPMENT	ESTABLISHMENT OF PROTEIN LOCALIZATION
TISSUE DEVELOPMENT	POSITIVE REGULATION OF RESPONSE TO STIMULUS	POSITIVE REGULATION OF BIOSYNTHETIC PROCESS	CELLULAR CATABOLIC PROCESS
INTRACELLULAR SIGNAL TRANSDUCTION	BIOLOGICAL ADHESION	INTRACELLULAR SIGNAL TRANSDUCTION	SMALL MOLECULE METABOLIC PROCESS
POSITIVE REGULATION OF MOLECULAR FUNCTION	POSITIVE REGULATION OF MOLECULAR FUNCTION	EMBRYO DEVELOPMENT	ORGANONITROGEN COMPOUND METABOLIC PROCESS
PHOSPHATE CONTAINING COMPOUND METABOLIC PROCESS	REGULATION OF INTRACELLULAR SIGNAL TRANSDUCTION	POSITIVE REGULATION OF RESPONSE TO STIMULUS	SINGLE ORGANISM CATABOLIC PROCESS
POSITIVE REGULATION OF CELL COMMUNICATION	VESICLE MEDIATED TRANSPORT	PHOSPHATE CONTAINING COMPOUND METABOLIC PROCESS	MACROMOLECULAR COMPLEX ASSEMBLY
REGULATION OF PHOSPHORUS METABOLIC PROCESS	ION TRANSPORT	ORGAN MORPHOGENESIS	ESTABLISHMENT OF LOCALIZATION IN CELL
HOMEOSTATIC PROCESS	INTRACELLULAR SIGNAL TRANSDUCTION	POSITIVE REGULATION OF MOLECULAR FUNCTION	PROTEIN COMPLEX SUBUNIT ORGANIZATION
REGULATION OF MULTICELLULAR ORGANISMAL DEVELOPMENT	TISSUE DEVELOPMENT	REGULATION OF PHOSPHORUS METABOLIC PROCESS	REGULATION OF CELLULAR RESPONSE TO STRESS
RESPONSE TO OXYGEN CONTAINING COMPOUND	PROTEIN LOCALIZATION	POSITIVE REGULATION OF CELL COMMUNICATION	SINGLE ORGANISM BIOSYNTHETIC PROCESS
CELLULAR RESPONSE TO ORGANIC SUBSTANCE	ORGAN MORPHOGENESIS	REGULATION OF RESPONSE TO STRESS	PHOSPHATE CONTAINING COMPOUND METABOLIC PROCESS
REGULATION OF RESPONSE TO STRESS	NEGATIVE REGULATION OF MOLECULAR FUNCTION	REGULATION OF PROTEIN MODIFICATION PROCESS	REGULATION OF RESPONSE TO STRESS
PROTEIN LOCALIZATION	REGULATION OF MULTICELLULAR ORGANISMAL DEVELOPMENT	CELL DEVELOPMENT	RESPONSE TO OXYGEN CONTAINING COMPOUND
REGULATION OF PROTEIN MODIFICATION PROCESS	LIPID METABOLIC PROCESS	REGULATION OF TRANSCRIPTION FROM RNA POLYMERASE II PROMOTER	ORGANIC ACID METABOLIC PROCESS
EMBRYO DEVELOPMENT	POSITIVE REGULATION OF CATALYTIC ACTIVITY	REGULATION OF CELL DEATH	PROTEIN COMPLEX BIOGENESIS
POSITIVE REGULATION OF GENE EXPRESSION	NEGATIVE REGULATION OF RESPONSE TO STIMULUS	CELLULAR RESPONSE TO ORGANIC SUBSTANCE	POSITIVE REGULATION OF CELLULAR COMPONENT ORGANIZATION
IMMUNE SYSTEM PROCESS	REGULATION OF CELL PROLIFERATION	ANATOMICAL STRUCTURE FORMATION INVOLVED IN MORPHOGENESIS	LIPID METABOLIC PROCESS
ANATOMICAL STRUCTURE FORMATION INVOLVED IN MORPHOGENESIS	CELLULAR RESPONSE TO ORGANIC SUBSTANCE	CELL DEATH	VESICLE MEDIATED TRANSPORT

(B)

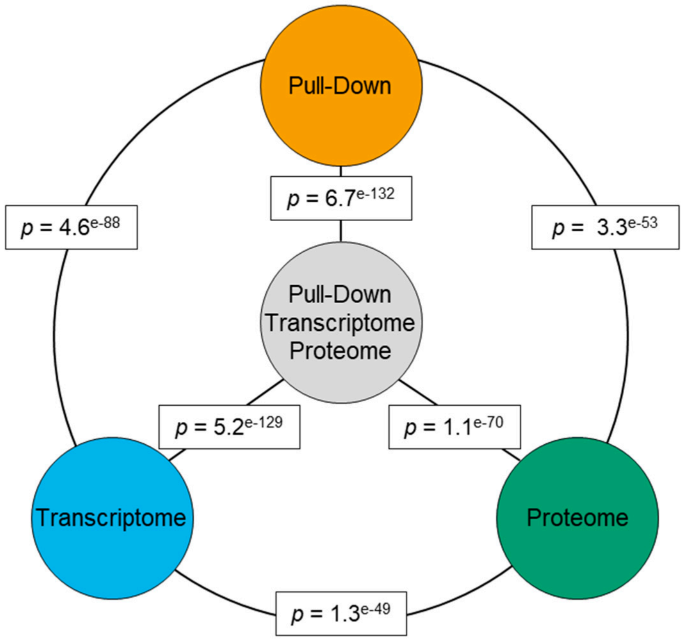

Figure S3. MiR-491-5p affects SKOV3 cells motility.
(**A**) Relative wound density in real time on Incucyte S3 system. Mean value +/- SD for triplicate points of a representative experiment. (**B**) Bar chart of wound density values 18 h after wound. Wound healing area was measured by Incucyte S3 software. (**C**) Representative pictures of wound areas just after wounding and at 6 h, 12 h and 18 h time points.

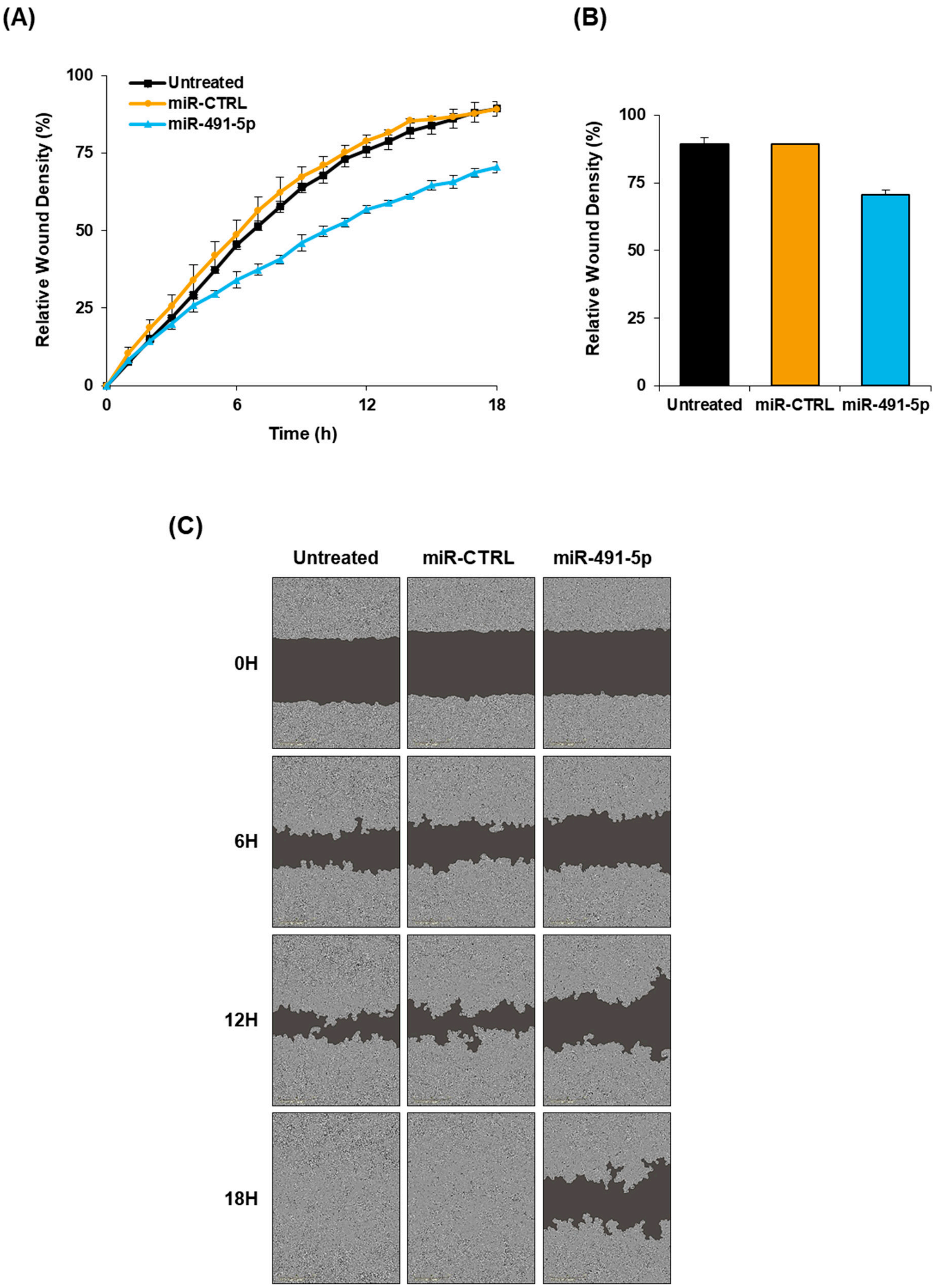

Figure S4. Raw blots from Figure 5

Original blots images are provided by the imager within a green frame. Samples related specifically to this figure part are with legends, samples unrelated to this specific figure part which were imaged at the same on different membranes are legended as « other ».

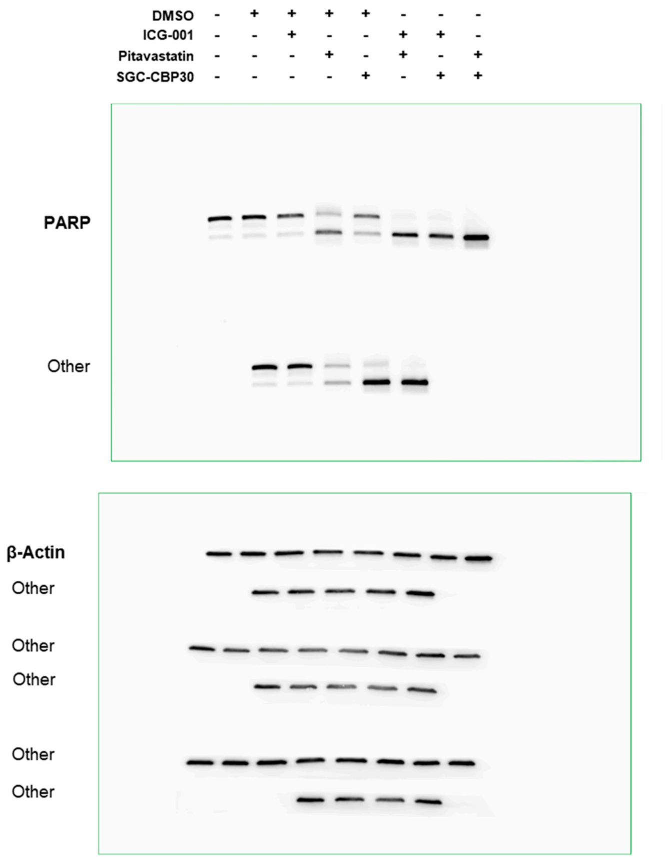

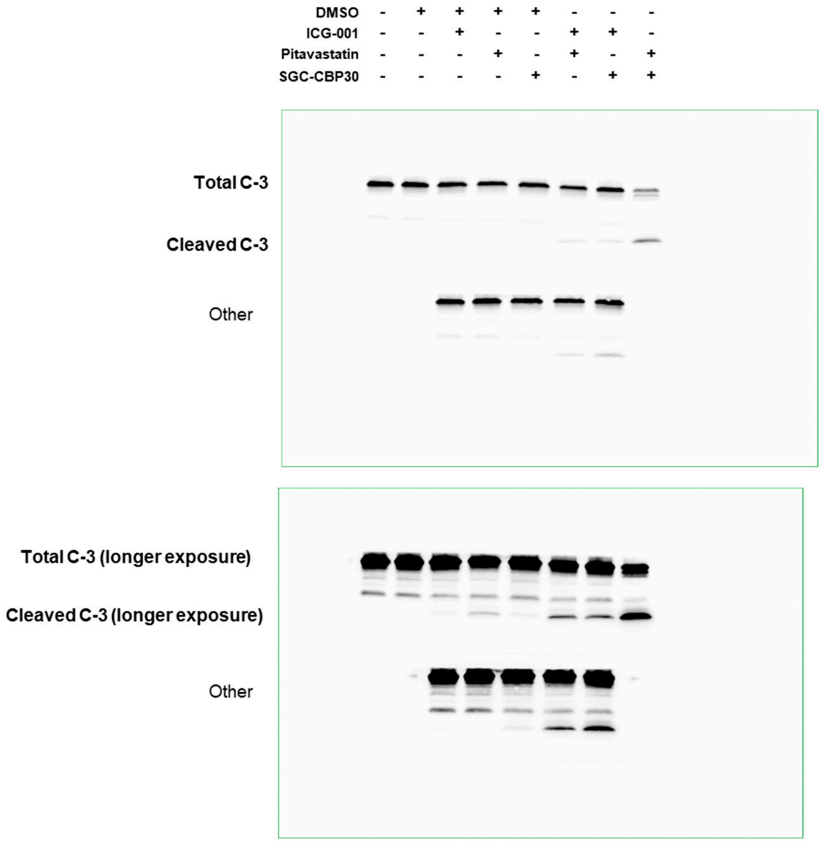

Figure S5. Effects of miR-491-5p (all cell lines) and cell count (IGROV1-R10) and CellTiter Glo (SKOV3 and OAW42-R) upon combined inhibition of selected hubs.

(A) Viable IGROV1-R10 cell count by Trypan blue exclusion method at indicated time points and after indicated treatments. Results are mean +/- SD **(B)** (**C**) and (**D**) Representative pictures of the cell layer and cell distribution into cell cycle 72 h after indicated transfections of IGROV1-R10 cells (**B**), SKOV3 cells (**C**) or OAW42-R cells (**D**). Condensed and fragmented DAPI-stained nuclei are typical of apoptosis. Flow cytometry results show, sub-G1 events represent fragmented nuclei and cell death. (**E**) and (**F**) Drug concentrations in cell culture media were as follow: ICG-001 = 25 µM, Pitavastatin = 10 µM and SGC-CBP30 = 15 µM. Because drugs were solubilized in DMSO, DMSO was added for single-drug conditions to achieve the same DMSO final concentration in each condition. Measurement of cell viability in indicated conditions by CellTiter Glo 72 h post-treatment in SKOV3 (**E**) and OAW42-R (**F**) cells.

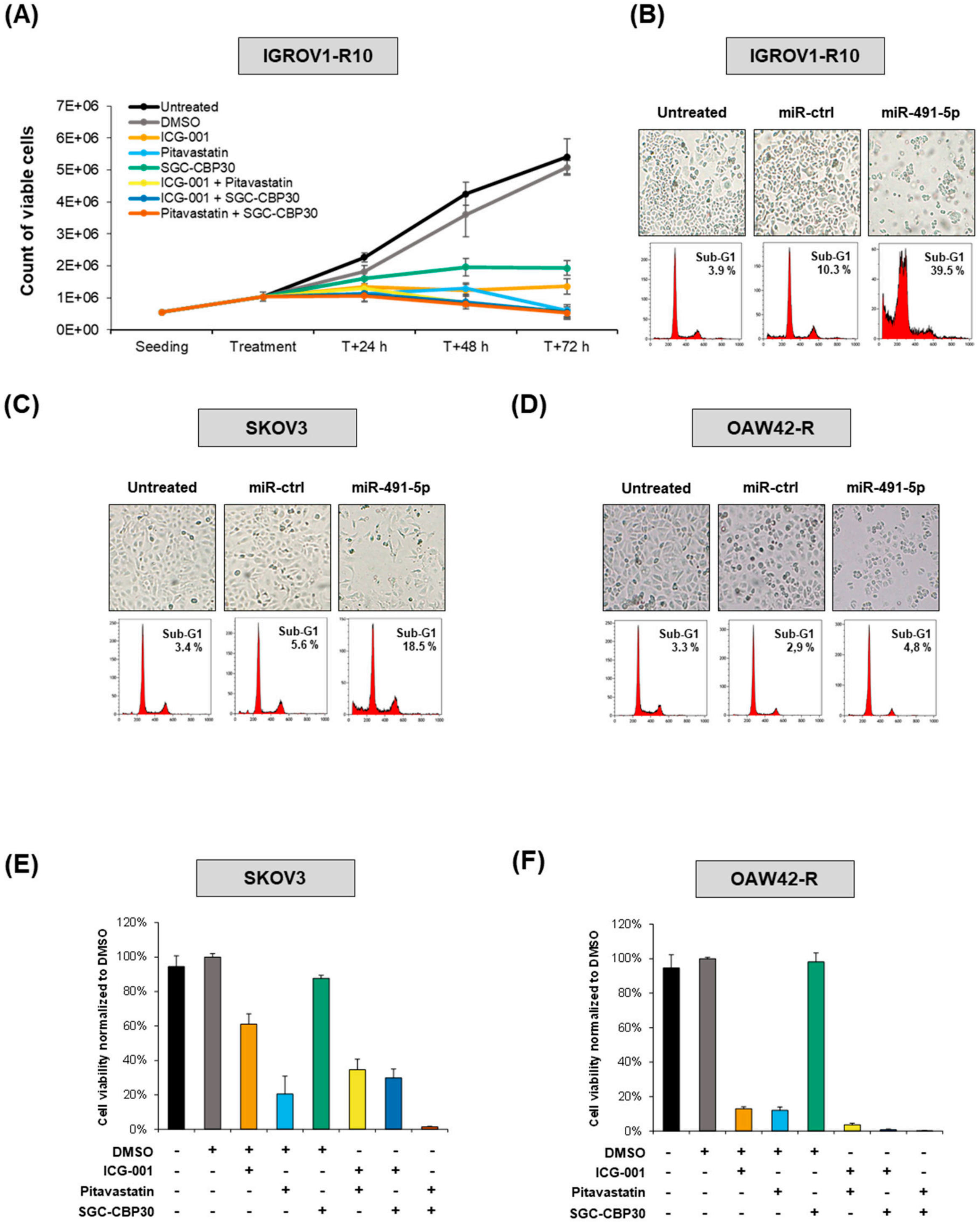

Supp Figure 6. Raw blots from Figure 6

Original blots images are provided by the imager within a green frame. Samples related specifically to this figure part are with legends, samples unrelated to this specific figure part which were imaged at the same on different membranes are legended as « other ».

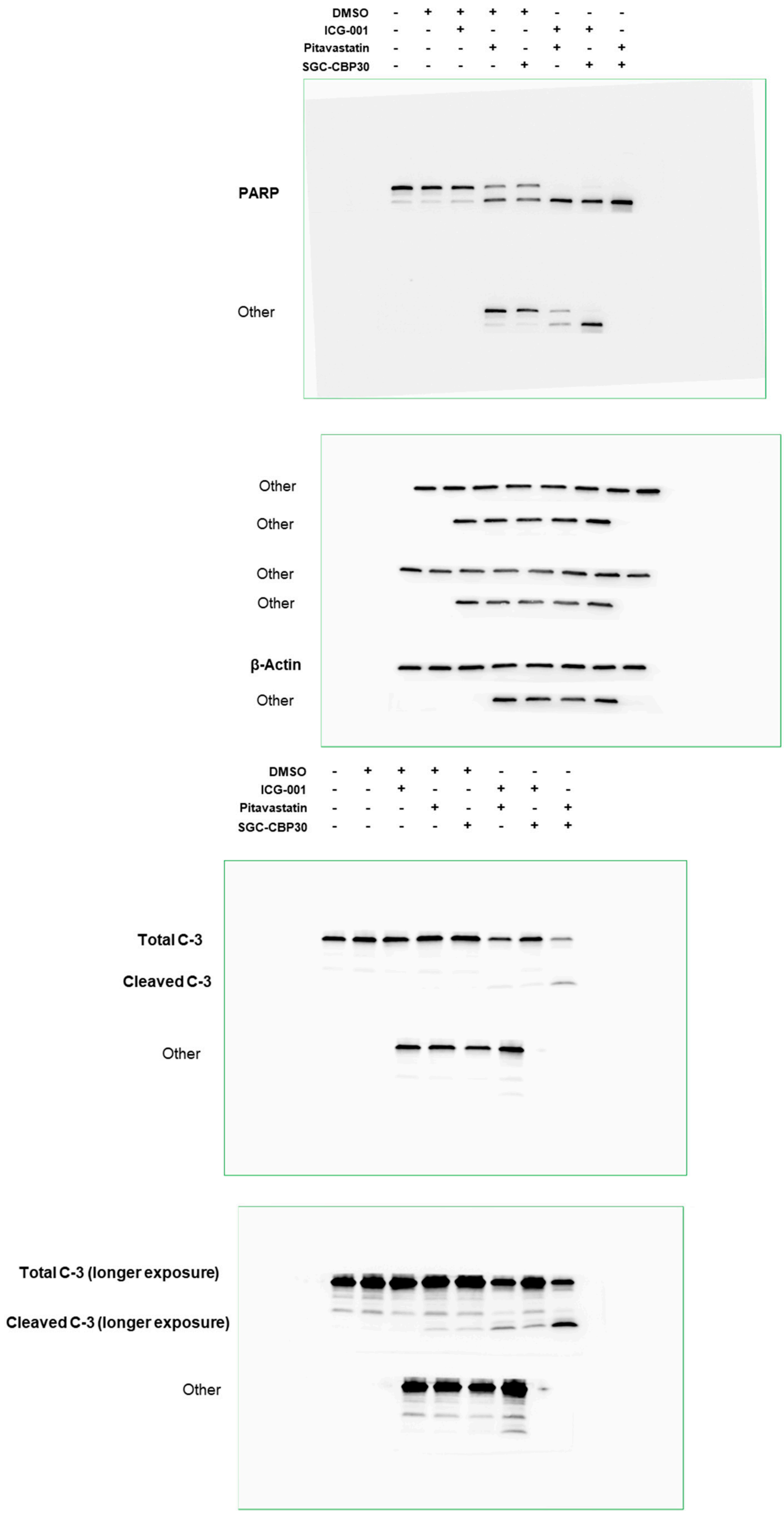

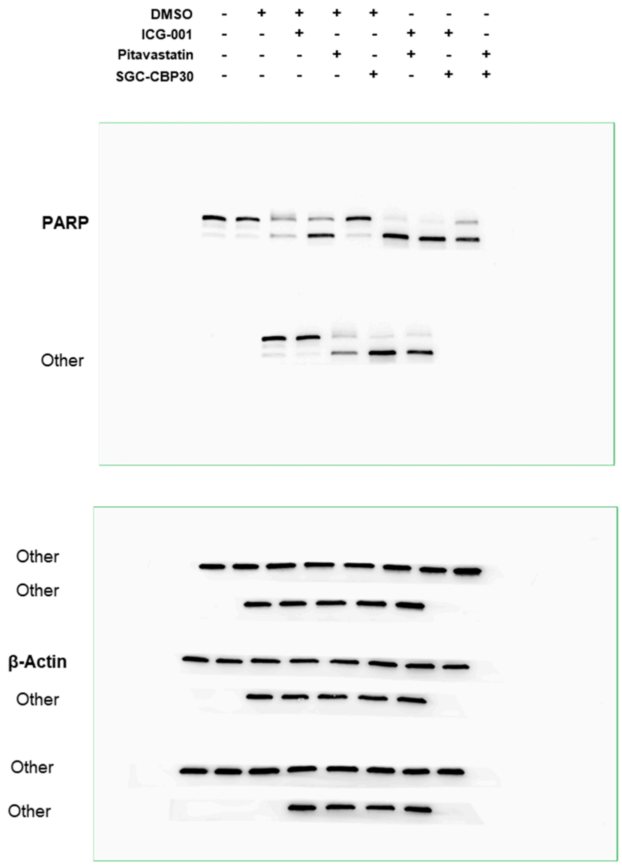
DMSO - + + + + - - -
ICG-001 - - + - - + + -
Pitavastatin - - - + - + - +
SGC-CBP30 - - - - + - + +
PARP
Other
Other
Other
β-Actin
Other
Other
Other

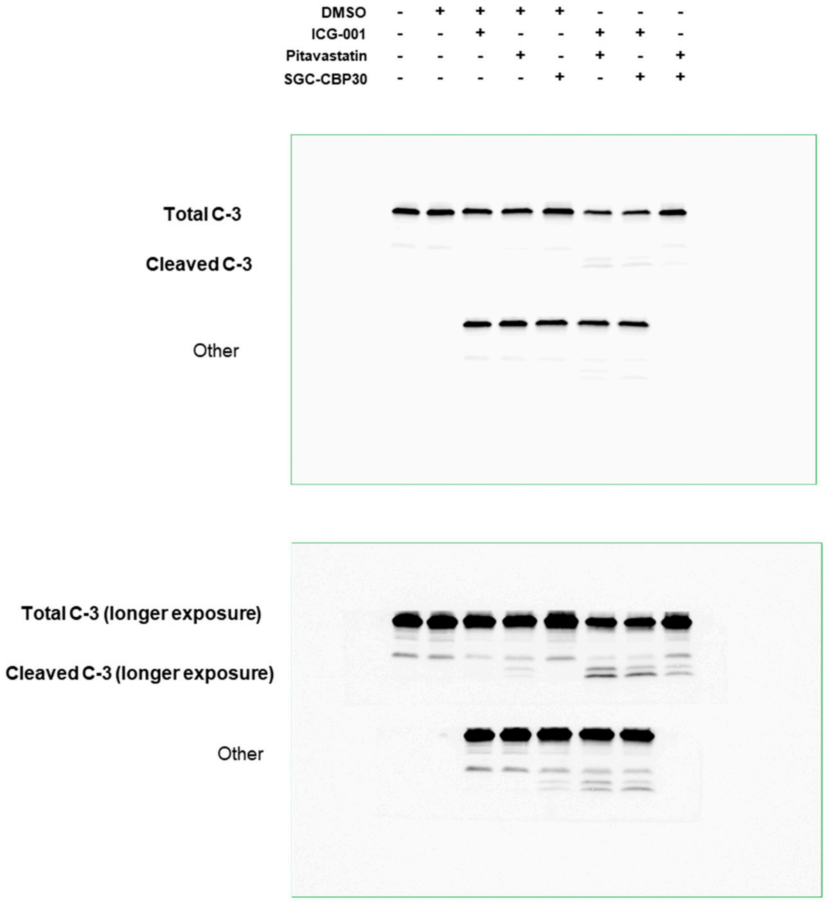
DMSO - + + + + - - -
ICG-001 - - + - - + + -
Pitavastatin - - - + - + - +
SGC-CBP30 - - - - + - + +
Total C-3
Cleaved C-3
Other
Total C-3 (longer exposure)
Cleaved C-3 (longer exposure)
Other

Supp Figure 7. Inhibition of selected hubs with Bcl-xL or EGFR inhibitor in IGROV1-R10 cells.
Drug concentrations in cell culture media were as follow: ICG-001 = 25 µM, Pitavastatin = 10 µM and SGC-CBP30 = 15 µM, ABT-737 = 5 µM, Gefitinib = 5 µM. Because drugs were solubilized in DMSO, DMSO was added for single-drug conditions to achieve the same DMSO final concentration in each condition. (**A**) and (**B**) Bar chart representing the cleaved caspase-3 activity relative to cell confluency 24 h, 48 h and 72 h post treatment, focused on ABT-737 (**A**) and Gefitinib (**B**) based combinations. Results are mean from 3 independent experiments.

(A)

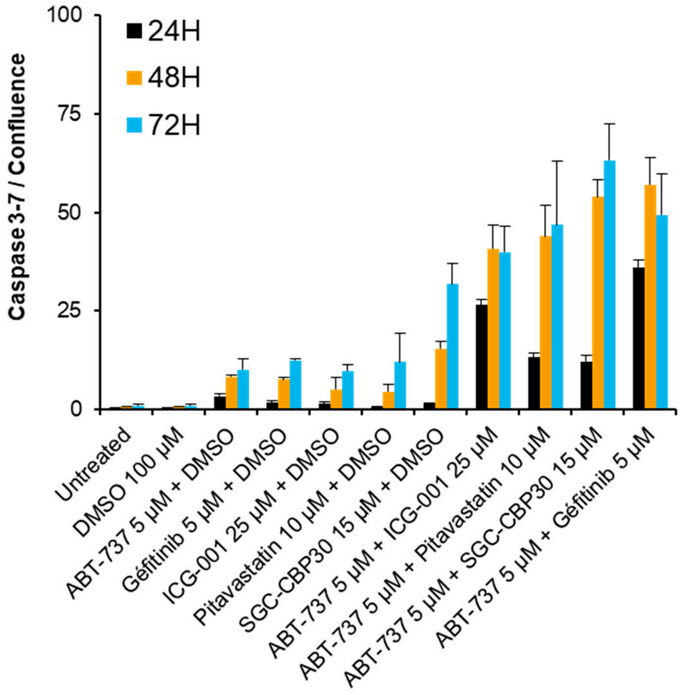

(B)

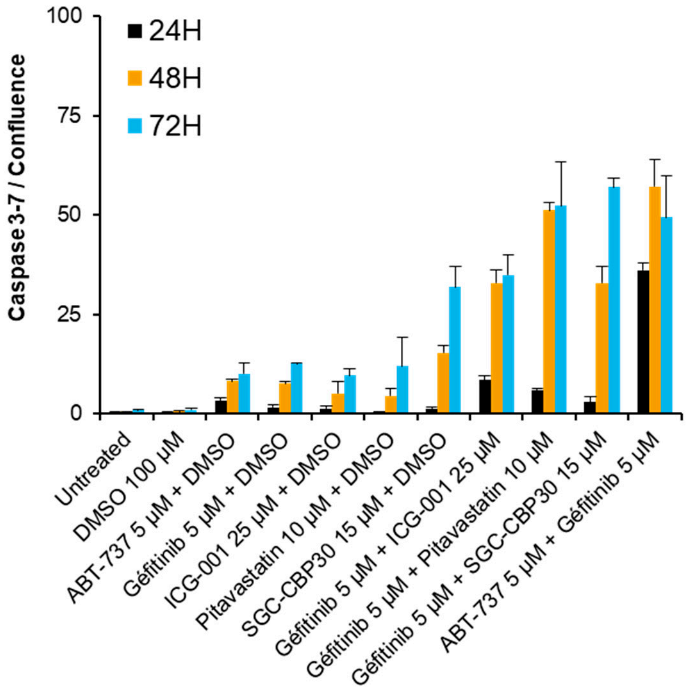

Table S1. Characteristics of the PPI networks.
Number of genes with at least 1 connection in the PPI network we constructed for the gene lists from each experiment or combination of experiments. The number of edges states for the total number of connections between genes in the corresponding PPI network. The average number of neighbors states for the average number of connections for each gene in the network.

Table S2. The identification of selected hubs requires combination of several datasets.
Recapitulates fold change values in log 2 scale from transcriptome, proteome and pull-down analysis for all selected hubs. RAC3, RHOB and RRAS are small GTPases. In bold, cut-off for fold-change/enrichment was reached for the corresponding experiment. ND: non-detected.

Table S3. Our combined experiment strategy identifies most of previously validated miR-491-5p direct targets.
Fold change values in log2 scale from transcriptome, proteome and pull-down analyses for all miR-491-5p validated direct targets from the literature. In bold, cut-off was reached for the corresponding analysis in our datasets. ND: Not Detected. KDM4B is also known as JMJD2B.

Dataset 1. Lists of enriched/differentially expressed genes selected from each experiment.

Dataset 2. Lists of genes in overlaps on common universes

Dataset 3. Lists of selected genes from individual and combined experiments.

Dataset 4. 100 most enriched GO-BP and NABA/REACTOME/KEGG/PID/BIOCARTA/ST pathways for individual and combined experiments.

Dataset 5. Hubs from the PPI network ranked according to the number of neighbors.

Dataset 6. Detailed characteristics of PPI networks
