# Supplementary figures and images for: Network-Based Integration of Multi-Omics Data Identifies the Determinants of miR-491-5p Effects
Source: Cancers (Basel). 2021 Aug 5;13(16):3970. doi: 10.3390/cancers13163970 (PMC8393872; doi:10.3390/cancers13163970)

(A)

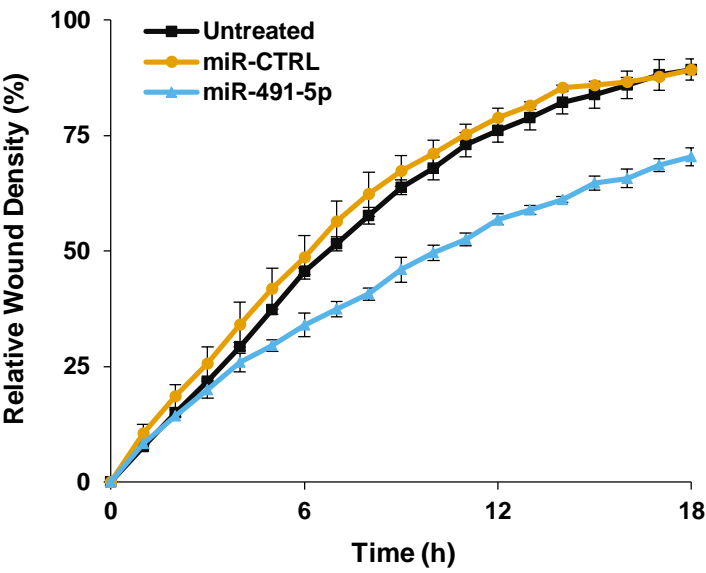

(B)

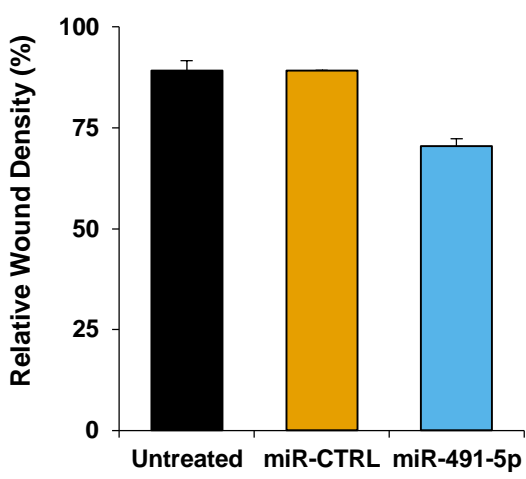

(C)

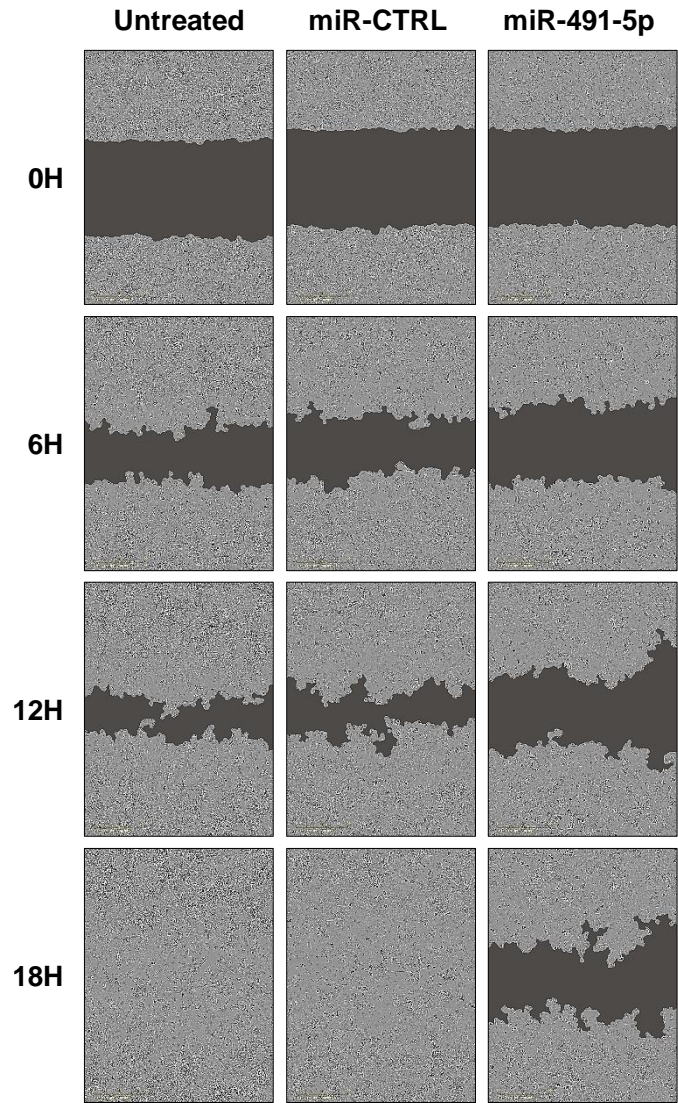

Supplement: Supplementary file 1 [file cancers-13-03970-s001.zip › Figure S3.pdf]

(A)

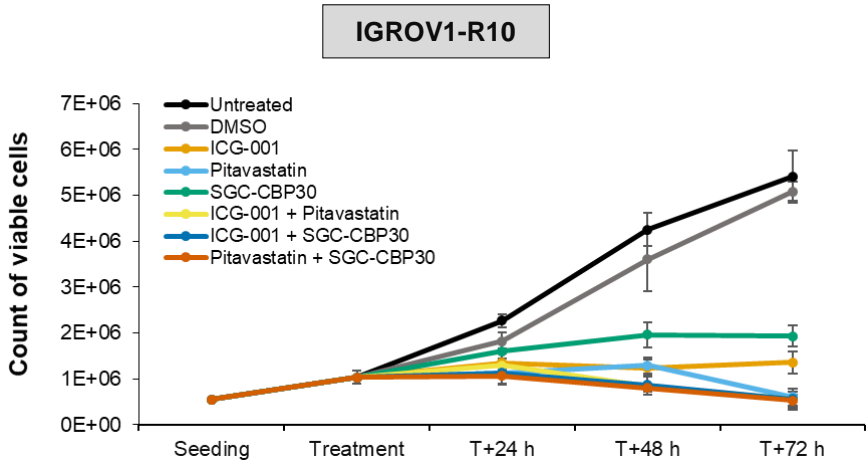

(B)

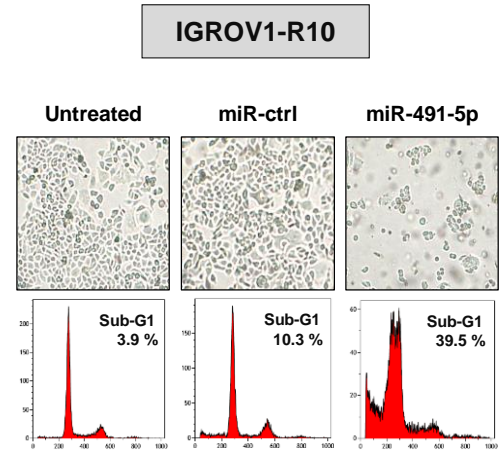

(C)

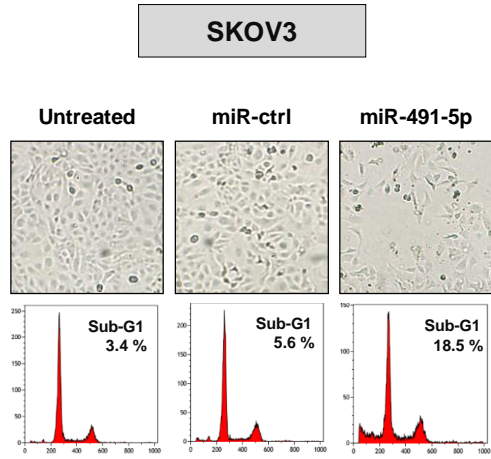

(D)

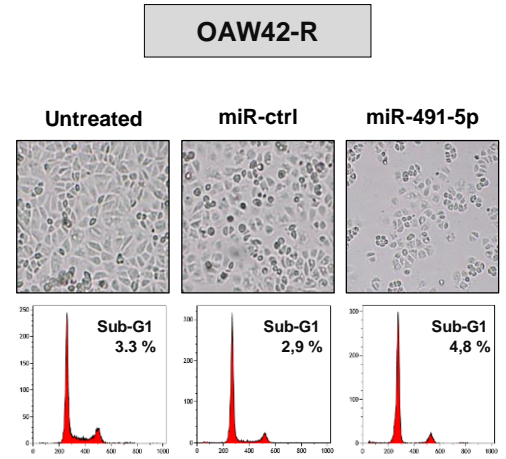

(E)

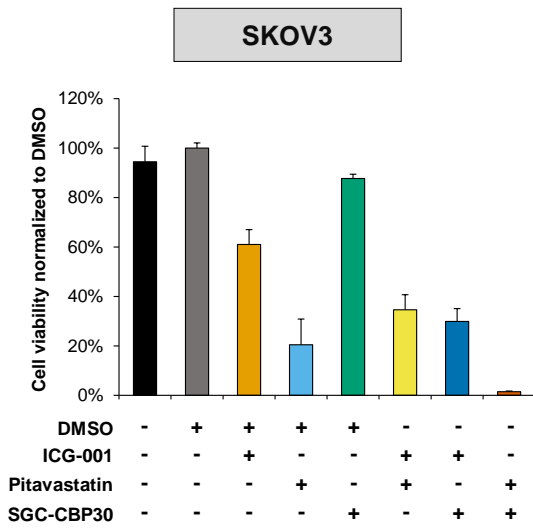

(F)

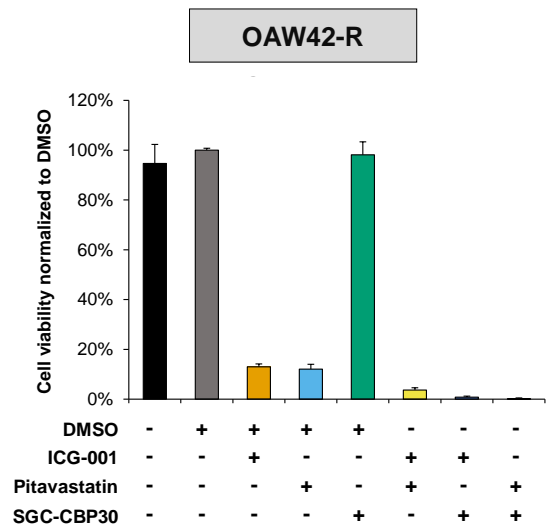

Supplement: Supplementary file 1 [file cancers-13-03970-s001.zip › Figure S5.pdf]

(A)

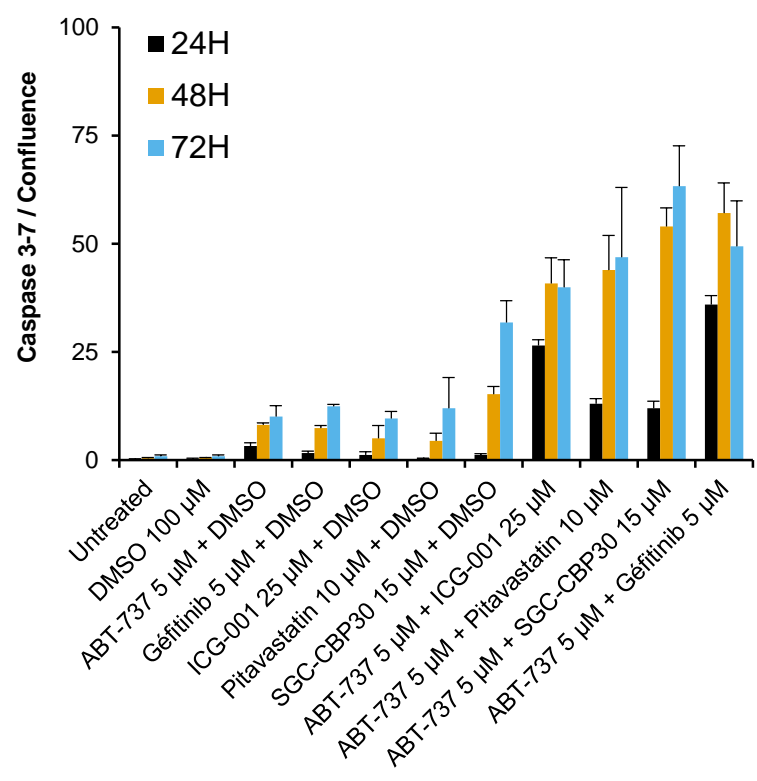

(B)

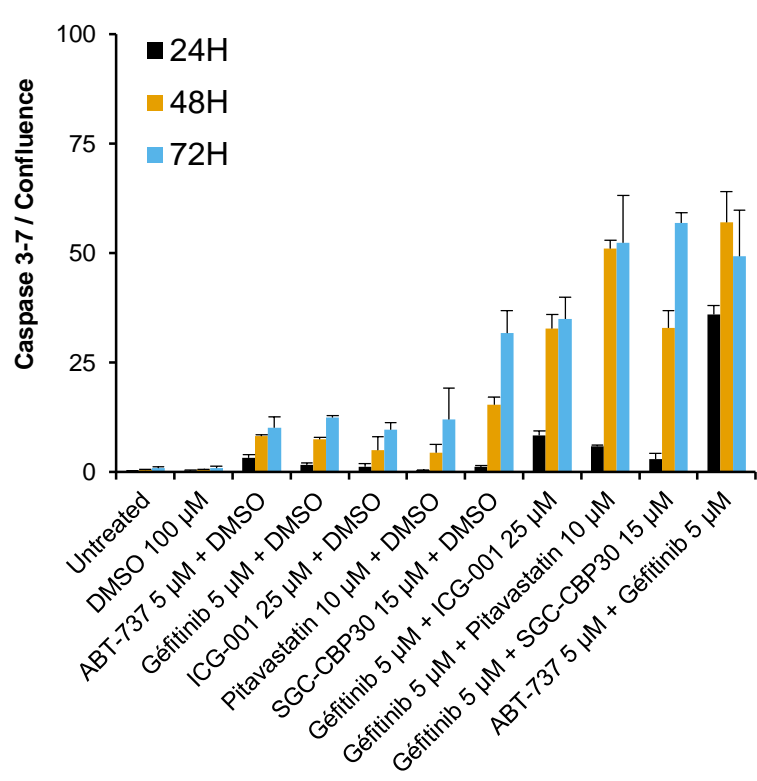

Supplement: Supplementary file 1 [file cancers-13-03970-s001.zip › Figure S7.pdf]
